# Metabifurcation analysis unveils hidden dynamical structure of a neural population model

**DOI:** 10.1186/1471-2202-12-S1-P197

**Published:** 2011-07-18

**Authors:** Federico Frascoli, Lennaert van Veen, Ingo Bojak, David TJ Liley

**Affiliations:** 1 Brain and Psychological Sciences Research Centre, Swinburne University of Technology, Hawthorn, Victoria 3122, Australia; 2 Faculty of Science, University of Ontario Institute of Technology, Oshawa, Ontario L1H 7K4, Canada; 3 Centre for Neuroscience, Donders Institute for Brain, Cognition and Behaviour, Radboud University Nijmegen Medical Centre, 6500 HB Nijmegen, The Netherlands

## 

In a forthcoming publication [1], we find links between the physiological parameters and dynamics of a neural population model (NPM), which are not accessible by conventional methods. They emerge only when parameter space is systematically partitioned according to bifurcation responses, a method we call “metabifurcation analysis”. This is made possible by an automated bifurcation analysis of a large sample of admissible parameter sets and generalizes straightforwardly to the investigation of other models with large and complex parameter spaces.

We study concretely the Liley NPM, which requires 29 different - biologically meaningful - parameters to be specified for bulk activity. In Ref. [2], 73,454 parameter sets were found that generate EEG power spectra predictions closely resembling those of human wakeful rest (“alpha sets”) at physiological neural firing rates. However, like many biologically realistic models (e.g., the Jansen-Rit NPM [3]), the Liley NPM supports a rich variety of dynamics which are generally distributed “fractally” over parameter space. Consequently, “alpha sets” are spread seemingly randomly throughout parameter space and hence a systematic classification of their dynamics and physiological correlations proves difficult.

By studying bifurcation diagrams of a sub-sample of 405 “alpha sets”, we discovered that without exception all of them could be classified into two distinct dynamical families with regards to a continuation in *R*, governing overall inhibitory synaptic strength, and *k*, the relative effect of inhibition on excitatory vs. inhibitory subpopulations. Essentially, Family 1 (F1) has two almost parallel saddle-node lines in the *R-k* plane, whereas Family 2 (F2) sports two cusp points organizing two saddle-node wedges. We then sorted all 73,454 “alpha sets” into F1 and F2, respectively, by an automated procedure using AUTO-07P [4] that examined these distinctive features.

Four important results were obtained by this bifurcation partitioning: 1) Reactions to the modeled application of the anesthetic agent isoflurane differ, with F2 being three times as likely as F1 to show strong, transient spectral power increases. 2) Exogenous stimuli modeled by thalamic input can control transitions between the dynamic families. 3) Distributions of the values of two excitatory population parameters differed strongly between F1 and F2, providing the potential for endogenous control. 4) The repertoire of (multi)stable oscillations for variations of inhibitory strength *R* was much more extensive for F2.

In summary, we showcase here a new method for partitioning the complex parameter spaces of biologically realistic models according to their bifurcation behavior: “metabifurcation analysis”. For the Liley NPM, we find that one can classify “alpha sets” into two dynamical families that one can speculatively consider as representing “explorative” (F2, multistable oscillatory dynamics) and “consolidating” (F1, simple noise filter dynamics) brain states, where transitions between F1 and F2 are possible via exogenous stimuli.
